# Adherence with direct oral anticoagulants in patients with atrial fibrillation: Trends, risk factors, and outcomes

**DOI:** 10.1002/joa3.12656

**Published:** 2021-11-18

**Authors:** Anat Arbel, Zomoroda Abu‐Ful, Meir Preis, Shai Cohen, Walid Saliba

**Affiliations:** ^1^ Department of Internal Medicine B Lady Davis Carmel Medical Center Haifa Israel; ^2^ Department of Community Medicine and Epidemiology Lady Davis Carmel Medical Center Haifa Israel; ^3^ Institute of Hematology Lady Davis Carmel Medical Center Haifa Israel; ^4^ Ruth and Bruce Rappaport Faculty of Medicine Technion‐Israel Institute of Technology Haifa Israel

**Keywords:** anticoagulants, atrial fibrillation, direct‐acting oral anticoagulants, treatment adherence

## Abstract

**Background:**

Adherence to direct oral anticoagulants (DOACs) remains a concern among non‐valvular atrial fibrillation (AF) patients. We aimed to assess patterns of adherence with DOACs and examine their association with ischemic stroke and systemic embolism (SE).

**Methods:**

This retrospective cohort study includes all adult members of Clalit Health Services, the largest healthcare provider in Israel, with newly diagnosed non‐valvular AF between January 2014 and March 2019, who initiated DOACs within 90 days of AF diagnosis and used DOACs exclusively. Adherence was assessed using the proportion of days covered (PDC) over the first year of treatment, and high adherence was defined as PDC ≥80%. Regression models were used to identify predictors of high adherence to DOACs and to examine the association between adherence and stroke or SE.

**Results:**

Overall 15,255 patients were included in this study. The proportion of highly adherent (PDC ≥80%) DOACs users was around 75% and decreased slightly over the years. On multivariable analyses, the likelihood of high adherence to DOACs increased with age and across higher socioeconomic classes, and was more likely among females, Jews, statins users, and patients with CHA_2_DS_2_‐VASc score ≥2. Risk of stroke and SE was lower among highly adherent DOACs users; adjusted HR 0.56 (95% CI, 0.45–0.71), compared to users with PDC <80%.

**Conclusions:**

Adherence with DOACs is still sub‐optimal among non‐valvular AF patients, resulting in a higher risk of stroke and SE.

## Graphical abstract

High adherence to DOACs reduces the risk of stroke and systemic thromboembolism. With approximately 75% of patients highly adhering to DOACs, adherence is suboptimal. Older age, higher socioeconomic class, female sex, Jewish ethnicity, and CHA2DS2‐VASc score≥2 are predictors of high adherence.

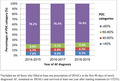

## 1 INTRODUCTION

Atrial fibrillation (AF) is a common cardiac rhythm disorder which poses a significant risk for cerebrovascular morbidity and mortality.^1^ When used appropriately, oral anticoagulants (OACs) have shown to reduce the incidence of embolic stroke in AF by more than 50%.^2^

Vitamin K antagonists (VKAs) have long been considered the main OAC used worldwide. VKAs have several limitations such as high bleeding risk, slow onset of action, the need for frequent monitoring, and numerous drug and diet interactions. Direct oral anticoagulants (DOACs) have been recently introduced and have been gradually replacing VKAs owing to their distinct benefits in terms of efficacy, safety, convenience, more predictable effect and fewer drug and diet interactions.^3^ However, their short half‐life time and their rapid onset of action require a strict treatment compliance and adherence in order to maintain the desirable antithrombotic effect.^4^, ^5^, ^6^

It has been shown that less than half of the patients adhere to OAC treatment over time.^7^ There was an expectation that DOACs introduction, given their advantages, would be translated into improved adherence. Unfortunately, adherence to DOACs remains poor in patients with AF,^7^, ^8^, ^9^, ^10^, ^11^, ^12^ and appears to be associated with increased risk of stroke,^11^, ^12^ yet real‐life data is scant.^7^, ^8^, ^9^, ^10^, ^11^, ^12^

Adherence patterns are an integral part of clinical decision making and depend on various factors, either social or clinical. Understanding those patterns and factors is clinically relevant and may be helpful in better resources utilization and health planning.

In this study, we use a population‐based real‐life data to assess patterns and trends of patients’ adherence with DOACs treatment. This study also aims to provide specific insight as for the implication of non‐adherence to DOACs on the risk of ischemic stroke and systemic embolism (SE).

## 2 METHODS

### 2.1 Source of data

This study is based on data from the computerized database of Clalit Health Services (CHS) which provides inclusive health care for more than half of the Israeli population. Health care coverage in Israel is mandatory according to the National Health Insurance Law (1995), and is provided by four groups akin to not‐for‐profit health maintenance organizations (HMO). All members of the different HMOs have a similar health insurance plan and similar access to health services, including low medications copayment. The electronic medical records (EMRs) of CHS include data from multiple sources: records of primary care physicians, community specialty clinics, hospitalizations, laboratories, and pharmacies. A registry of chronic diseases diagnoses is compiled from these data sources. Diagnoses are captured in the registry by diagnosis‐specific algorithms, employing International Classification of Diseases Ninth revision (ICD‐9) code reading, text reading, laboratory test results, and disease‐specific drug usage. A record is kept of the data sources and dates used to establish the diagnosis, with the earliest recorded date, from any source, considered to be the defining date of diagnosis. Several high‐quality population‐based studies have been conducted based on data retrieved from CHS database.^13^, ^14^

### 2.2 Study population

**FIGURE 1:**
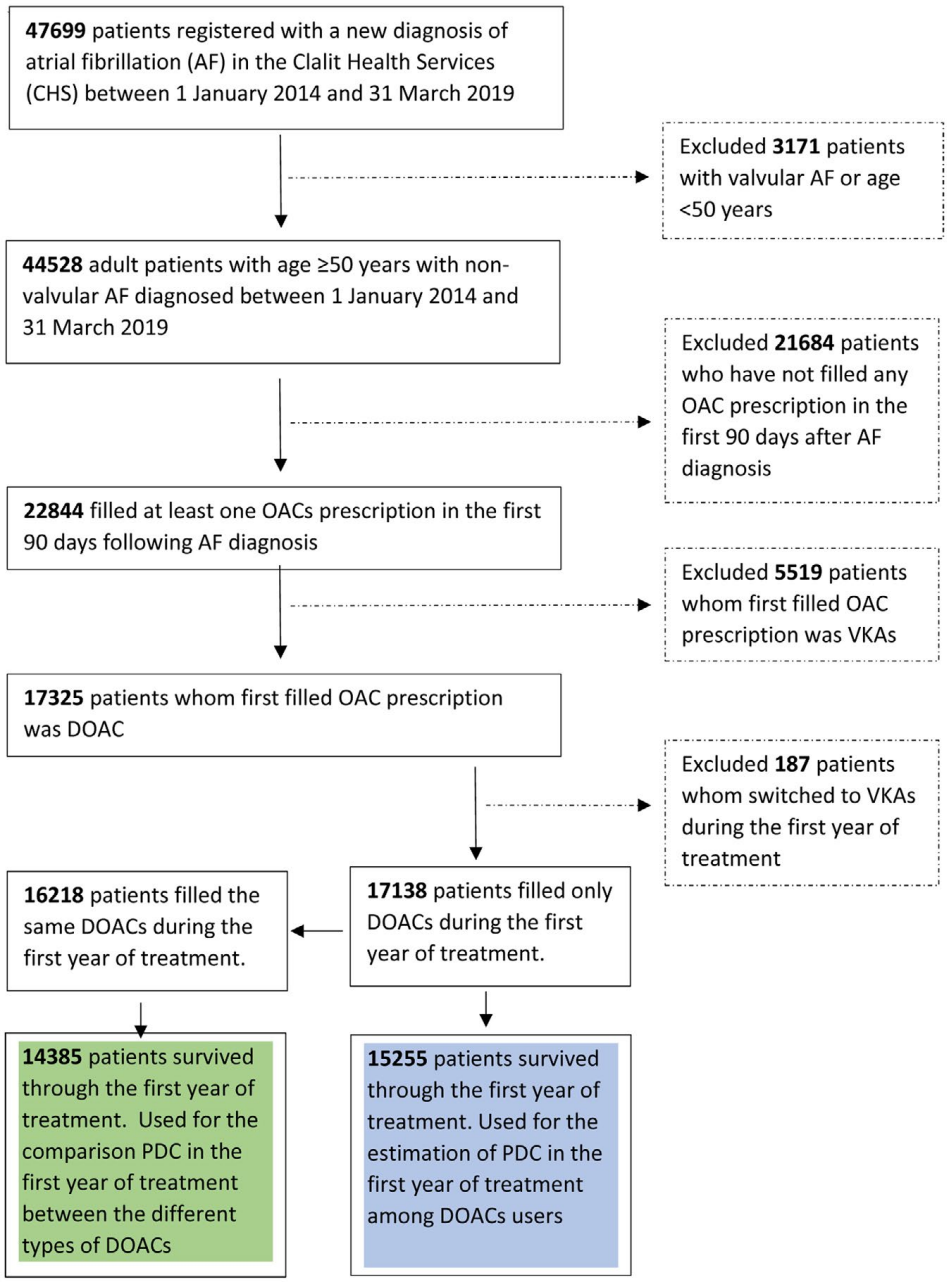
Flowchart depicting patients’ selection for studying the proportion of days covered (PDC) with DOACs

We used the computerized database of Clalit Health Services (CHS) to retrospectively identify all adults patients aged 50 years or older with newly diagnosed non‐valvular AF (ICD‐9, 427.31 and 427.32) between 1 January 2014 and 31 March 2019. For studying 1‐year adherence to DOACs treatment, we included patients who filled OAC prescription during the first 90 days after AF diagnosis, patients whose first OAC prescription was DOACs, patients who did not switched treatment from DOACs to VKAs during the first year of treatment and patients who survived for at least 12 months after first DOACs prescription date in order to keep homogeneity in the 1‐year adherence assessment. The date of first‐filled DOACs prescription constituted the index date. The flowchart in Figure 1 depicts the selection of the study population.

The study was approved by the Review Board of the Lady Davis Medical Centre and conducted in accordance with the Declaration of Helsinki.

### 2.3 OACs use

The use of OACs was determined based on CHS pharmacy records using ATC codes: B01AF01 (rivaroxaban), B01AF02 (apixaban), B01AE07 (dabigatran), and B01AA03 (warfarin). Data on medication use is considered to be complete because of the low copayment required in the CHS making it unlikely that prescription medications are purchased in non‐CHS pharmacies. Detailed prescription information on dates of prescriptions filled and number of treatment days supplied enabled us to estimate adherence.

DOACs were included in the Israeli national healthcare insurance plan before 2014. At the beginning, DOACs were supplied at low copayment for patients with non‐valvular AF and CHADS_2_ score ≥4 points. The indications for low copayment were updated in January 2014 to include non‐valvular AF patients with CHADS_2_ of ≥3, and later in January 2016 to include non‐valvular AF patients with CHADS_2_ of ≥2, and lastly in January 2019 to include non‐valvular AF patients with CHADS_2_ of ≥1.

### 2.4 Study outcomes and definition of terms

Adherence to DOACs was assessed using the proportion of days covered (PDC), as recommended by the Pharmacy Quality Alliance (PQA).^15^ PDC is calculated by dividing the number of days with treatment supply during the follow‐up period by the number of follow‐up days. In our study, we examined the 1‐year PDC starting from the index date. For DOACs prescriptions extending beyond 1 year, we included only the supply for days within the first year of treatment. High adherence with DOACs was defined as PDC ≥80%.^15^

We assessed the association between adherence and the composite outcome of ischemic stroke and SE, defined as a primary discharge diagnosis of ischemic stroke (ICD‐9; 433.X1, 434.X1, 436) and SE (ICD‐9; 444.X, 445.X).

### 2.5 Covariates

Demographic characteristics, and data on comorbidities were retrieved from the CHS‐computerized database for the calculation of CHA_2_DS_2_‐VASc score, a widely used risk stratification score for stroke prediction in patients with AF.^16^ In addition, we searched the CHS‐computerized database for socioeconomic status (SES) defined based on the SES score of the clinic neighborhood as defined by the Israeli Central Bureau of Statistics, smoking (ever versus never), statins use, and fecal occult blood test (FOBT) performed within the prior 2 years. SES had missing values, hence we used it as a categorical variable that included a category of missing values.

### 2.6 Statistical methods

Statistical analyses were performed in 2020–2021 using IBM SPSS Statistics 24.0 (IBM). For all analyses, *p* < .05 for the two‐tailed tests was considered statistically significant. Continuous variables are summarized with means and standard deviations (SD), and categorical variables are summarized with counts and proportions.

Multivariable logistic regression using backward selection was used to identify predictors independently associated with increased adherence to anticoagulants (PDC ≥80%).

Kaplan–Meier curves were used to depict the distribution of time to composite of stroke or SE. Cox proportional hazard regression models were used to estimate the hazard ratios (HRs) and the 95% confidence interval (95% CI) for the association between PDC and stroke or SE. For this analysis the follow‐up started by the end of the 12 months required for PDC estimation and extended until the event of interest (stroke or SE), death or the end of follow‐up in 30 September 2020, whichever came first. PDCs were modeled as binary variable (PDC ≥80% *vs*. PDC <80%), as ordinal variable (PDC <40%, PDC 40%–60%, PDC 60%–80%, and PDC ≥80%) with PDC <40% serving as reference category, and as continuous variable to estimate the HR for each 10% increase in PDC. Multivariable models that were used to assess the association between PDC and ischemic stroke or SE were adjusted for CHA_2_DS_2_‐VASc score.

## 3 RESULTS

### 3.1 Adherence patterns

Overall, 44 528 patients were newly diagnosed with non‐valvular AF between January 2014 and March 2019. Of them, 15 255 patients were early initiators and exclusively DOACs users and survived through the first year of treatment. We used this group of patients for the estimation of 1‐year PDC. Of these patients, 14,385 used the same DOAC during the first year of treatment, hence were used for the comparison of 1‐year PDC between the different types of DOACs (Figure 1). The distribution of demographic and clinical baseline characteristics of these two groups of patients, stratified by three time periods (2014–2015, 2016–2017, 2018–2019) of AF diagnosis, is depicted in Tables 1 and 2. The distribution of some baseline characteristics showed statistically significant differences between the three time periods. However, except for a tendency of increasing apixaban use and decreasing frequency of the individual components of CHA_2_DS_2_‐VASc score through the years, those differences were mild and did not seem to hold clinical significance.

**TABLE 1:** Baseline characteristics of 14,385 patients who filled at least one prescription of DOACs in the first 90 days of newly diagnosed AF, remained on the same type of DOAC and survived at least 1 year after starting treatment, stratified by the year of atrial fibrillation diagnosis

Variable	All (*n* = 14 385)	Year of atrial fibrillation diagnosis			*p* value
		2014–2015 (*n* = 3108)	2016–2017 (*n* = 6482)	2018–2019 (*n* = 4795)	
Age (years)	77.0 ± 9.3	77.7 ± 9.0	77.0 ± 9.3	76.5 ± 9.6	<.001
Age categories					<.001
<65 years	1634 (11.4%)	312 (10.0%)	722 (11.1%)	600 (12.5%)	
65–75 years	3940 (27.4%)	700 (22.5%)	1780 (27.5%)	1460 (30.4%)	
≥75 years	8811 (61.3%)	2096 (67.4%)	3980 (61.4%)	2735 (57.0%)	
Sex					.905
Males	6524 (45.4%)	1412 (45.4%)	2927 (45.2%)	2185 (45.6%)	
Females	7861 (54.6%)	1696 (54.6%)	3555 (54.8%)	2610 (54.4%)	
Ethnicity					<.001
Jews	12977 (90.2%)	2900 (93.3%)	5860 (90.4%)	4217 (87.9%)	
Arabs	1408 (9.8%)	208 (6.7%)	622 (9.6%)	578 (12.1%)	
Socioeconomic status					<.001
Low	4459 (31.0%)	866 (27.9%)	2014 (31.1%)	1579 (32.9%)	
Middle	6437 (44.7%)	1432 (46.1%)	2899 (44.7%)	2106 (43.9%)	
High	3298 (22.9%)	747 (24.0%)	1482 (22.9%)	1069 (22.3%)	
FOBT (prior 2 years)	3423 (23.8%)	667 (21.5%)	1570 (24.2%)	1186 (24.7%)	.002
eGFR ≥60 ml/min	7892 (54.9%)	1612 (51.9%)	3588 (55.4%)	2692 (56.1%)	<.001
Comorbidities					
CHF	2859 (19.9%)	630 (20.3%)	1250 (19.3%)	978 (20.4%)	.280
Diabetes	6675 (46.4%)	1530 (49.2%)	2988 (46.1%)	2157 (45.0%)	.001
Hypertension	12428 (86.4%)	2779 (89.4%)	5634 (86.9%)	4015 (83.7%)	<.001
Vascular diseases	4709 (32.7%)	1159 (37.3%)	2099 (32.4%)	1451 (30.3%)	<.001
Previous stroke/TIA	3576 (24.9%)	992 (31.9%)	1494 (23.0%)	1090 (22.7%)	<.001
Smoking	5425 (37.7%)	1147 (36.9%)	2403 (37.1%)	1875 (39.1%)	.051
DOAC type					<.001
Apixaban	9635 (67.0%)	1660 (53.4%)	4524 (69.8%)	3451 (72.0%)	
Dabigatran	1293 (9.0%)	293 (9.4%)	599 (9.2%)	401 (8.4%)	
Rivaroxaban	3457 (24.0%)	1155 (37.2%)	1359 (21.0%)	943 (19.7%)	
CHA_2_DS_2_‐VASc score	4.40 ± 1.65	4.72 ± 1.61	4.36 ± 1.61	4.24 ± 1.70	<.001

Note

SES was missing in 191 (1.3%) of patients; eGFR was missing in 999 (6.9%) of patients.

Abbreviations: CHF, congestive heart failure; eGFR, estimated glomerular filtration rate; FOBT, fecal occult blood in stool test; VKAs, vitamin K antagonists.

**TABLE 2:** Baseline characteristics of 15,255 patients who filled at least one prescription of DOACs in the first 90 days of newly diagnosed AF and survived at least one year after starting treatment, stratified by the year of atrial fibrillation diagnosis

Variable	All (*n* = 15 255)	Year of atrial fibrillation diagnosis			*p* value
		2014–2015 (*n* = 3284)	2016–2017 (*n* = 6883)	2018–2019 (*n* = 5088)	
Age (years)	77.0 ± 9.3	77.7 ± 8.9	77.0 ± 9.3	76.5 ± 9.6	<.001
Age categories					<.001
<65 years	1703 (11.2%)	325 (9.9%)	757 (11.0%)	621 (12.2%)	
65–75 years	4227 (27.7%)	759 (23.1%)	1899 (27.6%)	1569 (30.8%)	
≥75 years	9325 (61.1%)	2200 (67.0%)	4227 (61.4%)	2898 (57.0%)	
Sex					.699
Males	6905 (45.3%)	1490 (45.4%)	3091 (44.9%)	2324 (45.7%)	
Females	8350 (54.7%)	1794 (54.6%)	3792 (55.1%)	2764 (54.3%)	
Ethnicity					<.001
Jews	13782 (90.3%)	3067 (93.4%)	6227 (90.5%)	4488 (88.2%)	
Arabs	1473 (9.7%)	217 (6.6%)	656 (9.5%)	600 (11.8%)	
Socioeconomic status					<.001
Low	4728 (31.0%)	915 (27.9%)	2144 (31.1%)	1669 (32.8%)	
Middle	6821 (44.7%)	1517 (46.2%)	3072 (44.6%)	2232 (43.9%)	
High	3509 (23.0%)	788 (24.0%)	1575 (22.9%)	1146 (22.5%)	
FOBT (prior 2 years)	3635 (23.8%)	715 (21.8%)	1670 (24.3%)	1250 (24.6%)	.007
eGFR ≥60 ml/min	8387 (55.0%)	1711 (52.1%)	3819 (55.5%)	2857 (56.2%)	<.001
Comorbidities					
CHF	3011 (19.7%)	665 (20.2%)	1315 (19.1%)	1031 (20.3%)	.205
Diabetes	7053 (46.2%)	1623 (49.4%)	3153 (45.8%)	2277 (44.8%)	<.001
Hypertension	13180 (86.4%)	2939 (89.5%)	5988 (87.0%)	4253 (83.6%)	<.001
Vascular diseases	4984 (32.7%)	1231 (37.5%)	2223 (32.3%)	1530 (30.1%	<.001
Previous stroke/TIA	3777 (24.8%)	1040 (31.7%)	1586 (23.0%)	1151 (22.6%)	<.001
Smoking	5746 (37.7%)	1204 (36.7%)	2552 (37.1%)	1990 (39.1%)	.031
CHA_2_DS_2_‐VASc score	4.39 ± 1.64	4.72 ± 1.61	4.36 ± 1.60	4.23 ± 1.69	<.001

Note

SES was missing in 197 (1.3%) of patients; eGFR was missing in 1048 (6.9%) of patients.

Abbreviations: CHF, congestive heart failure; eGFR, estimated glomerular filtration rate; FOBT, fecal occult blood in stool test; VKAs, vitamin K antagonists.

**FIGURE 2:**
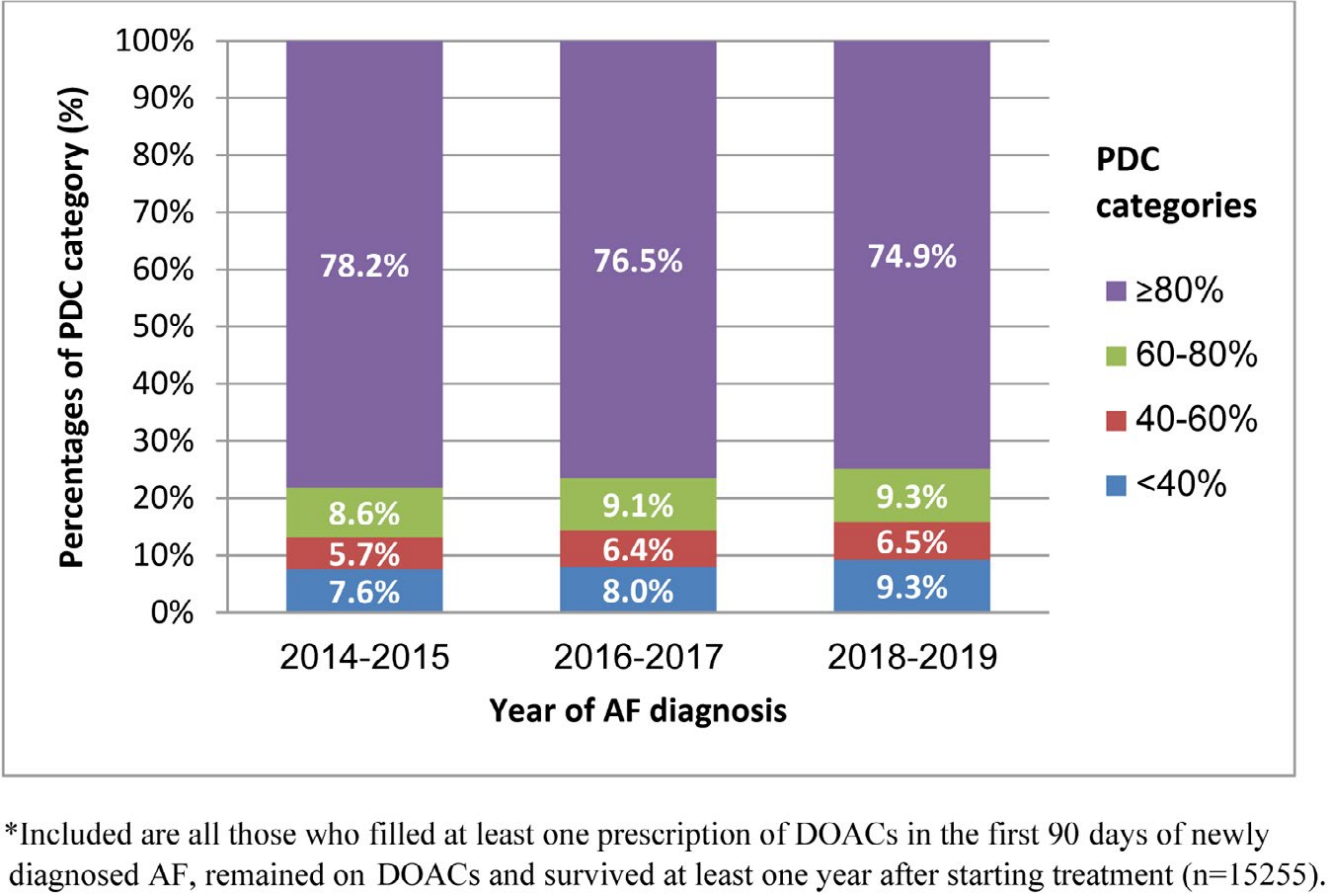
The distribution of DOACs’ PDC as estimated in the first year of treatment among newly diagnosed AF patients, stratified by the year of AF diagnosis*

**FIGURE 3:**
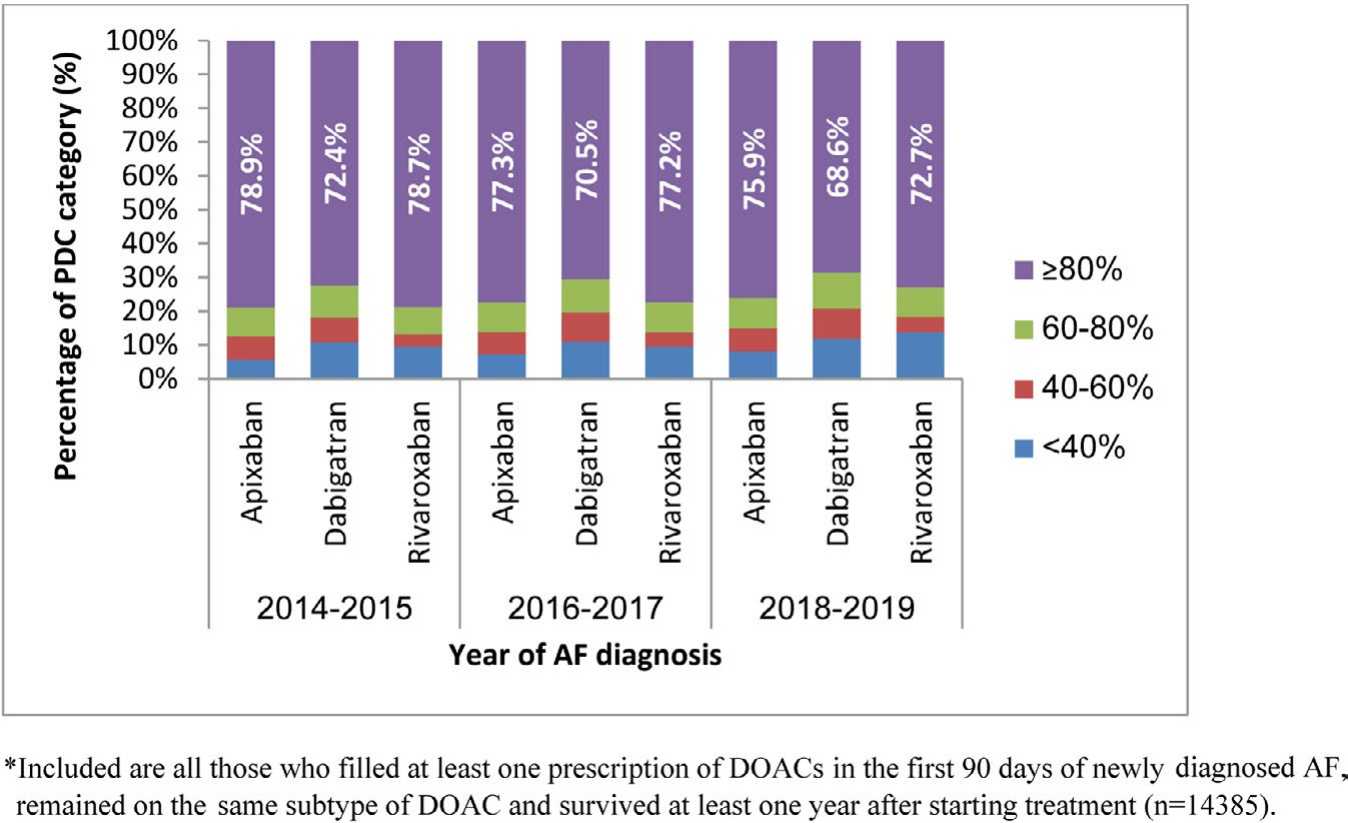
The distribution of DOACs’ PDC as estimated in the first year of treatment among newly diagnosed AF patients, compared between DOACs types and stratified by the year of AF diagnosis*

The distribution of 1‐year PDC to DOACs, stratified by year of AF diagnosis, is shown in Figure 2. The proportion of patients with high adherence to DOACs, defined as PDC ≥80%, slightly decreased over time; 78.2% in 2014–2015, 76.5% in 2016–2017, and 74.9% in 2018–2019 (Figure 2). A similar gradual decrease was demonstrated separately for each DOACs type as well. Apixaban and rivaroxaban users showed slightly higher proportions of high adherence to treatment, compared to dabigatran users (Figure 3).

### 3.2 Predictors of high adherence to DOACs

Multivariable logistic regression analysis revealed that the likelihood of high adherence to DOACs (PDC ≥80%), during the first year of treatment, increased with age and with increasing socioeconomic classes, and was more likely among females compared to males, among Jews compared to Arabs, among patients treated with statins and among patients with CHA_2_DS_2_‐VASc score ≥2 compared to those with CHA_2_DS_2_‐VASc score <2. The likelihood of high adherence was lower in smokers compared to nonsmokers. Compared to rivaroxaban use, the likelihood of high adherence was significantly lower with dabigatran use, whereas no statistically significant difference was observed with apixaban use (Table 3).

**TABLE 3:** Predictors of high adherence to DOACs defined as PDC ≥80% in the first years of treatment^a^

Variable	Multivariable^b^ Odds Ratio (OR) and 95% confidence interval (95% CI)
Age (years)	
<65	Reference
65–75	1.41 (1.23–1.61)
≥75	1.71 (1.50–1.94)
Sex	
Males	Reference
Females	1.17 (1.07–1.28)
Ethnicity	
Arabs	Reference
Jews	1.99 (1.75–2.27)
Socioeconomic class (SES)	
Low	Reference
Middle	1.29 (1.17–1.41)
High	1.61 (1.43–1.81)
Smoking (ever)	0.88 (0.81–0.96)
Previous treatment with statins	1.72 (1.59–1.86)
DOAC subtype	
Rivaroxaban	Reference
Apixaban	0.96 (0.87–1.06)
Dabigatran	0.78 (0.68–0.91)
CHA_2_DS_2_‐VASc score	
<2 points	Reference
≥2 points	1.66 (1.35–2.05)

^a^ Included are newly diagnosed AF who filled at least one DOACs prescription in the first 90 days of AF diagnosis (as first OAC treatment), remained in the same DOAC, and survived at least one year after starting treatment (*n* = 14385).

^b^ Multivariable logistic regression using backward selection was used to identify independent predictors of PDC ≥80%. The following variables were included in the model: age (3 categories), sex, ethnicity (Jews vs. Arabs), socioeconomic status (SES), and smoking (ever vs. never), FOBT for CRC screening in the last two years, statins use as a proxy of previous preventive treatment use, year of AF diagnosis, eGFR (>60 vs. ≥60 ml/min), DOAC subtype (apixaban vs. rivaroxaban vs. dabigatran), and CHA_2_DS_2_‐VASc score (≥2 vs. <2 points).

### 3.3 Association between adherence and stroke or SE

**FIGURE 4:**
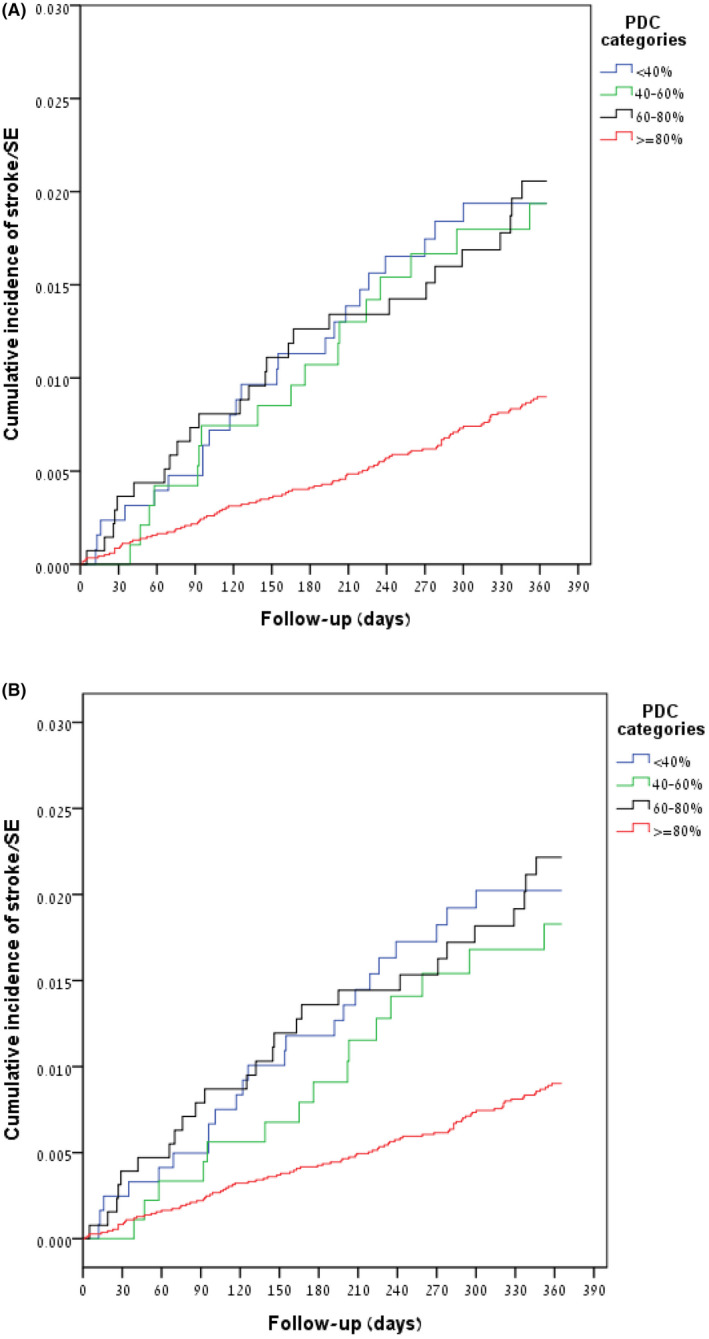
Kaplan–Meier curves depicting the distribution of time to stroke and systemic thromboembolism (SE), by PDC categories, in the first year after the end of the year following the first DOACs prescription date (a period that was used to estimate PDC). (A) All patients on DOACS (*n* = 15 255), and (B) patients who remained on the same DOAC (*n* = 14 385)

The risk of ischemic stroke and SE, after adjustments to CHA_2_DS_2_‐VASc score, was found to be 44% lower (adjusted HR 0.56, 95% CI 0.45–0.71) among highly adherent DOACs users (PDC ≥80%) compared to DOACs users with PDC <80% (Table 4). Using PDC as a ordinal variable with the lowest category (PDC <40%) serving as reference category, it was shown that increasing PDC up to 80% was not associated with stroke and SE risk reduction, and that only high adherence (PDC ≥80%) was associated with a statistically significant decrease of stroke and SE; adjusted HR 0.59 (95% CI, 0.42–0.83) (Table 4 and Figure 4). A protective effect was also demonstrated when using PDC as a continuous variable as well, with a 9% decrease (adjusted HR 0.91, 95% CI 0.88–0.95) in the risk of stroke and SE for each 10% increase in PDC (Table 4).

**TABLE 4:** Multivariable^a^ hazard ratios (HRs) for the association between DOACs use adherence, as estimated by PDC in the first years of treatment, and the risk of ischemic stroke and systemic thromboembolism

PDC category	All patients^b^ treated with DOACs (*n* = 15 255) Adjusted HR (95% CI)	Patients^c^ treated with the same DOAC (*n* = 14 385) Adjusted HR (95% CI)
PDC 2 categories		
PDC <80%	Reference	Reference
PDC ≥80%	0.56 (0.45–0.71)	0.56 (0.44–0.71)
PDC 4 categories		
PDC <40%	Reference	Reference
PDC 40–60%	1.12 (0.71–1.76)	1.10 (0.69–1.76)
PDC 60–80%	1.04 (0.68–1.58)	1.06 (0.69–1.63)
PDC ≥80%	0.59 (0.42–0.83)	0.59 (0.42–0.84)
PDC continuous variable *(HR for each 10% increase)*	0.91 (0.88–0.95)	0.91 (0.88–0.95)

^a^ Adjusted for the following variables: age (3 categories), sex, ethnicity (Jews vs. Arabs), socioeconomic status (SES), and smoking (ever vs. never), FOBT for CRC screening in the last two years, statins use, year of AF diagnosis, eGFR (>60 vs. ≥60 ml/min), CHA_2_DS_2_‐VASc score (ordinal variable), and DOAC subtype (only for the analysis of 14385 patients treated with same DOAC).

^b^ Included are all those who filled at least one prescription of DOACs in the first 90 days of newly diagnosed AF, and survived at least one year after starting treatment (*n* = 15255).

^c^ Included are all those who filled at least one prescription of DOACs in the first 90 days of newly diagnosed AF, remained on the same subtype of DOAC and survived at least one year after starting treatment (*n* = 14385).

## 4 DISCUSSION

This is one of the few large‐scale population‐based studies to assess adherence to DOACs. In this study, we found that approximately 75% of incident AF patients treated with DOACs were highly adherent to treatment, namely, were covered at least 80% of the days during the first year of treatment. The proportion of high adherence to DOACs in this study is consistent with the rates described in two recent large cohort studies conducted in France and Canada.^17^, ^18^

Nevertheless, as much as these results seem encouraging compared to previous studies,^7^, ^8^, ^9^ and despite the growing use of DOACs, non‐adherence to DOACs is still a concern, with the rate reaching up to 25% over a year period. Moreover, there is a worrisome progressive trend toward decreasing adherence to DOACs over the years. This trend can be explained, at least in part, by more extensive DOACs treatment because of the gradual broadening of indications for DOACs’ low copayment by Israeli health services, reflected in decreasing mean CHA_2_DS_2_‐VASc scores among their users over the years.

Higher adherence rates among older ages, among higher socioeconomic classes, among women, among Jews and among statin users, as well as lower rates among smokers, stand in line with results from other studies.^11^, ^17^, ^18^, ^19^ These findings can be explained by both patient's factors, including better health self‐care, health literacy, and trust in medical care; and treatment's factors, meaning its side effects. Of note, cost considerations are irrelevant because of low copayment of DOACs in Israel. Higher adherence among patients with CHA_2_DS_2_‐VASc score ≥2 was demonstrated previously,^18^ suggesting that symptomatic conditions and other comorbidities may result in stricter adherence. Those predictors may enable us to identify and implement focused interventions among potential non‐adherent DOACs users.

An important contribution of this study is providing an insight as for the implication of adherence level, estimated by PDC calculation, on adverse outcomes, using a real‐life data. In our study, high adherence to DOACs significantly reduced the risk of ischemic stroke and SE. While most studies demonstrated the protective effect of DOACs by comparing DOACs treated patients to warfarin‐treated patients^20^, ^21^, ^22^ or to antiplatelet agents treated patients,^23^ only few studies examined the association between their adherence and stroke.^11^, ^12^ The 9% decrease in the risk of stroke or SE for each 10% increase in PDC shown in our study, was slightly higher than the 7% decrease in the risk of stroke or death for each 10% increase in PDC, demonstrated in a small 2017 study among dabigatran users only.^11^ However, direct comparisons to those studies are difficult because of methodological differences.

A notable strength of this study is being a population‐based study with a relatively large number of AF patients. The fact that healthcare services in Israel are public and medication copayment is low makes the documentation of medications prescribing and purchases, reliable. In addition, relying on these documentations rather than patients’ self‐reports avoids the potential recall bias.

Nevertheless, our study is subject to some limitations. First, as an observational study, in which data is extracted from electronic health records, it is prone to misclassification bias or lacks data. Second, we relied on prescriptions and purchases data rather than actual taking the drugs by the patients. Thus, there might be an overestimation of adherence rates in cases in which patients stopped taking DOACs even though they had purchased the drugs. In addition, patients could have taken lower doses than purchased, hence being exposed to higher embolic risk. Finally, our study is prone to the healthy adherer effect.^24^ In other words, patients who are adherent to DOACs, may also adhere to other therapies as well as to healthier lifestyle and to medical preventive services, providing another explanation for the lower risk of embolic complications.

## 5 CONCLUSIONS

Adherence with DOACs treatment is still far from optimal, resulting in a substantial higher risk of embolic complications. More efforts should be done in order to increase physicians’ and AF patients’ awareness of the importance of compliance to DOACs.

## CONFLICTS OF INTEREST

The authors declare no conflicts of interest for this article.

## ETHICS APPROVAL

The study was approved by the Review Board of the Lady Davis Medical Centre and conducted in accordance with the Declaration of Helsinki.

## Data Availability

The data that support the findings of this study are available on request from the corresponding author. The data are not publicly available because of privacy or ethical restrictions.
